# Identification and Characterization of Sex-Biased MicroRNAs in *Bactrocera dorsalis* (Hendel)

**DOI:** 10.1371/journal.pone.0159591

**Published:** 2016-07-21

**Authors:** Wei Peng, Kaleem Tariq, Junfei Xie, Hongyu Zhang

**Affiliations:** State Key Laboratory of Agricultural Microbiology, Key Laboratory of Horticultural Plant Biology, Ministry of Education and Institute of Urban and Horticultural Entomology, College of Plant Science and Technology, Huazhong Agricultural University, Wuhan, Hubei, People’s Republic of China; Kunming University of Science and Technology, CHINA

## Abstract

MicroRNAs (miRNAs) are a class of endogenous small non-coding RNAs that regulate various biological processes including sexual dimorphism. The oriental fruit fly *Bactrocera dorsalis* is one of the most destructive agricultural insect pests in many Asian countries. However, no miRNAs have been identified from the separate sex and gonads to elucidate sex gonad differentiation in *B*. *dorsalis*. In this study, we constructed four small RNA libraries from whole body of females, males (except ovaries and testes) and ovaries, testes of *B*. *dorsalis* for deep sequencing. The data analysis revealed 183 known and 120 novel miRNAs from these libraries. 18 female-biased and 16 male-biased miRNAs that may be involved in sexual differentiation were found by comparing the miRNA expression profiles in the four libraries. Using a bioinformatic approach, we predicted *doublesex (dsx)* as a target gene of the female-biased miR-989-3p which is considered as the key switch gene in the sex determination of tephritid insects. This study reveals the first miRNA profile related to the sex differentiation and gives a first insight into sex differences in miRNA expression of *B*. *dorsalis* which could facilitate studies of the reproductive organ specific roles of miRNAs.

## Introduction

Sexual dimorphism is prevalent in insects, and it is thought that, transcriptional and post-transcriptional regulators have an impact in this biological process [1,2]. The regulation of sex-biased genes expression between males and females through several classes of small RNAs play important role in the sexual differentiation [3–5]. MicroRNAs (miRNAs) are a class of approximately 22 nucleotide (nt) endogenous non-coding small RNAs generated by Dicer enzymes processing of hairpin precursors that are involved in regulation gene expression at the post-transcriptional level [1,6–8]. In insects, mature miRNAs are loaded with an Argonaute protein to repress mRNA transcript or protein translation by binding to the 3’ UTR region of the target gene with imperfect complementarity [7]. The seed region (bases 2–8 from the 5’ end), which is the most highly conserved sequences of the miRNA contributes significantly to miRNA-target interaction [9–11]. The complex relationship between miRNA and mRNA form an intricate epigenetic mechanism for spatial and temporal gene expression regulation [12,13]. Thus miRNAs participate in regulating various biological processes including embryonic development, sexual identity, metamorphosis, fat metabolism and immune [3,14–17].

Recently, application of deep sequencing technology and in silico analysis have revealed amounts of miRNAs in plants, animals and viruses. The establishment of small RNA libraries of different life stages and specific tissue in these species stimulate a comprehensive and more in-depth studies of miRNAs in the development and other physiological process. The expression profiles and sex-biased miRNAs in sexual dimorphism have been characterized in the animals of mouse, chicken [18,19] and insects including *Drosophila melanogaster*, *Tribolium castaneum*, *Manduca sexta* [3,4,20,21]. Different miRNA expression profiles between testis and ovary in mouse have found 49 and 48 miRNAs exclusively existed in the gonad respectively. Mir-17-92 and Mir-106b-25 are involved in the mice spermatogenesis [18]. In *D*. *melanogaster*, sex-biased miRNAs are connected with the reproductive function and some new miRNAs are preferentially expressed in testis [4]. Furthermore, *let-7* miRNA was found as a somatic systemic signal modulators to establish and maintain sexual identity in sexs and differentiation in gonads [3]. In the nondrosophilid insect *T*. *castaneum*, oocytes miRNAs play important role in maternal transcript degradation process [21]. All these diverse miRNAs between sexs highlight the significant function role in germline development and sexual differentiation.

*Bactrocera dorsalis* (Hendel) (the oriental fruit fly), which is a badly invasive pest because of the damage to masses of fruits and vegetables like the citrus and guava, spread all over the South-East Asia and a number of Pacific Islands [22]. The fully sequenced genome and transcriptome analyses provide a greatly foundation database to understand the genetic network and molecular mechanism in *B*. *dorsalis *[23,24]. Although the previous report have sequenced the miRNAs during different developmental stages in life cycle of *B*. *dorsalis* [25] and different developmental stages of *B*. *dorsalis* testes [26], the miRNAs functions in sex determination and differentiation remain largely unknown.

In the present study, we constructed and sequenced four small RNA libraries between each sex and gonads of mature adult *B*. *dorsalis*, and identified numerous known and novel miRNAs highly expressed in the gonads, in order to understand these sex-biased miRNAs roles in reproduction and regulation of sex determination in *B*. *dorsalis*. These specific expression data will provide new informations to elucidate the regulatory role of miRNAs in sexually dimorphic traits and might contribute to understanding the sex determination mechanism in *B*. *dorsalis*.

## Results

### Global analysis of small RNA libraries

**Fig 1:**
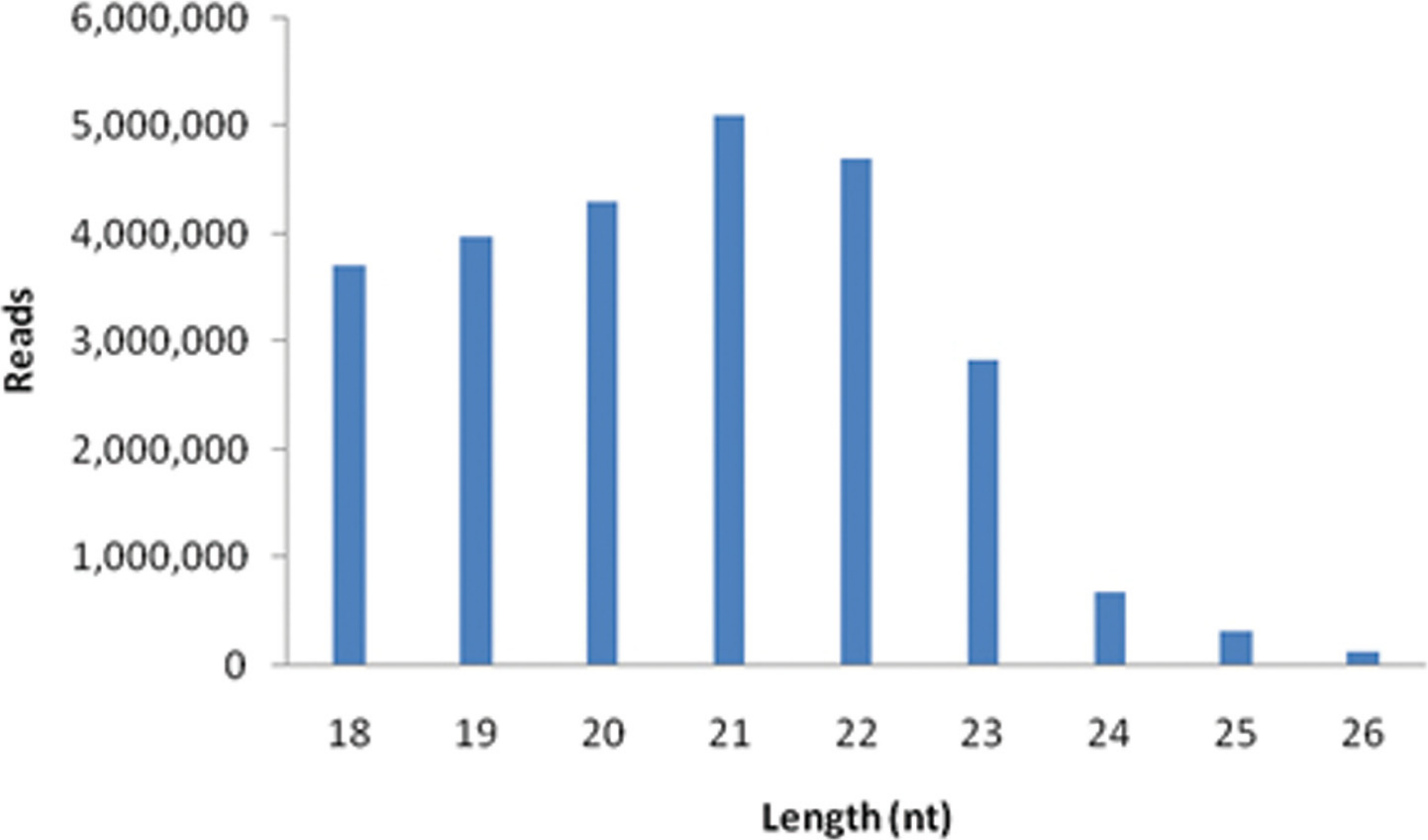
Length distribution and abundance of combined small RNAs in four libraries.

To identify the sex-biased miRNAs in *B*. *dorsalis*, four small RNA libraries from the mature females, males (without ovaries and testes) and ovaries, testes were constructed by using Illumina Solexa high-throughput sequencing technology. The four libraries produced a dataset of 40,931,727 raw reads in total: 10,482,821; 10,641,766 reads from the females and males. 9,989,208; 9,817,932 reads from the ovaries and testes (Table 1). These raw reads have been submitted to the National Center for Biotechnology Information (NCBI) Gene Expression Omnibus (accession number: GSE80536). After removing junk sequences, simple sequences, sequences longer than 26 nt or shorter than 18 nt and filtering the RFam (rRNA, tRNA, snRNA, snoRNA, and other RfamRNAs), *B*. *dorsalis* mRNAs and Repbase sequences, a total of 6,210,904; 7,237,349; 5,445,095 and 6,894,823 for females, males, ovaries and testes mappable clean reads were gained, respectively (Table 1). The length distribution of the total small RNA reads in the data set showed the majority of the small RNAs ranged from 19 nt to 22 nt and a peak at 21nt, which is the typical length of the mature miRNAs (Fig 1). Each sample’s length distribution showed in S1 Fig.

**Table 1:** Distribution of sequenced reads from raw data to cleaned sequences.

type	females		ovaries		males		testes	
	Total	%	Total	%	Total	%	Total	%
Raw reads	10,482,821	100	9,989,208	100	10,641,766	100	9,817,932	100
3ADT&length filter	2,811,145	26.82	3,686,552	36.91	1,956,525	18.39	1,583,814	16.13
Junk reads	33,056	0.32	12,664	0.13	19,497	0.18	18,282	0.19
Rfam	1,408,998	13.44	833,784	8.35	1,402,723	13.18	1,298,205	13.22
Repeats	21,617	0.21	12,658	0.13	28,793	0.27	25,562	0.26
rRNA	1,139,864	10.87	734,069	7.35	1,105,241	10.39	1,008,233	10.27
tRNA	119,424	1.14	35,617	0.36	118,800	1.12	127,510	1.30
snoRNA	9,531	0.09	13,203	0.13	7,776	0.07	8,387	0.09
snRNA	18,542	0.18	7,275	0.07	6,084	0.06	7,155	0.07
other Rfam RNA	121,637	1.16	43,620	0.44	164,822	1.55	146,920	1.50
Clean reads	6,210,904	59.25	5,445,095	54.51	7,237,349	68.01	6,894,823	70.23

### Identification of known and novel miRNAs from *B*. *dorsalis*

By filtering the data set and blasting against the known mature miRNAs and miRNA precursors in miRBase 20.0 (http://www.mirbase.org), we identified 141 miRNAs in the females library, 145 miRNAs in the males library, 127 miRNAs in the ovaries library, 143 miRNAs in the testes library and a total of 183 miRNAs in the combined data set (S1 Table). Low-energy, fold-back structures, and 18–24 nt mature miRNAs of miRNA precursors distinguish miRNAs from other small RNAs. The name of the most reads miRNA represented the particular miRNA and other variants. The expression levels of *B*. *dorsalis* known miRNAs ranging from 404222.33 counts for the most abundant to single count.

**Fig 2:**
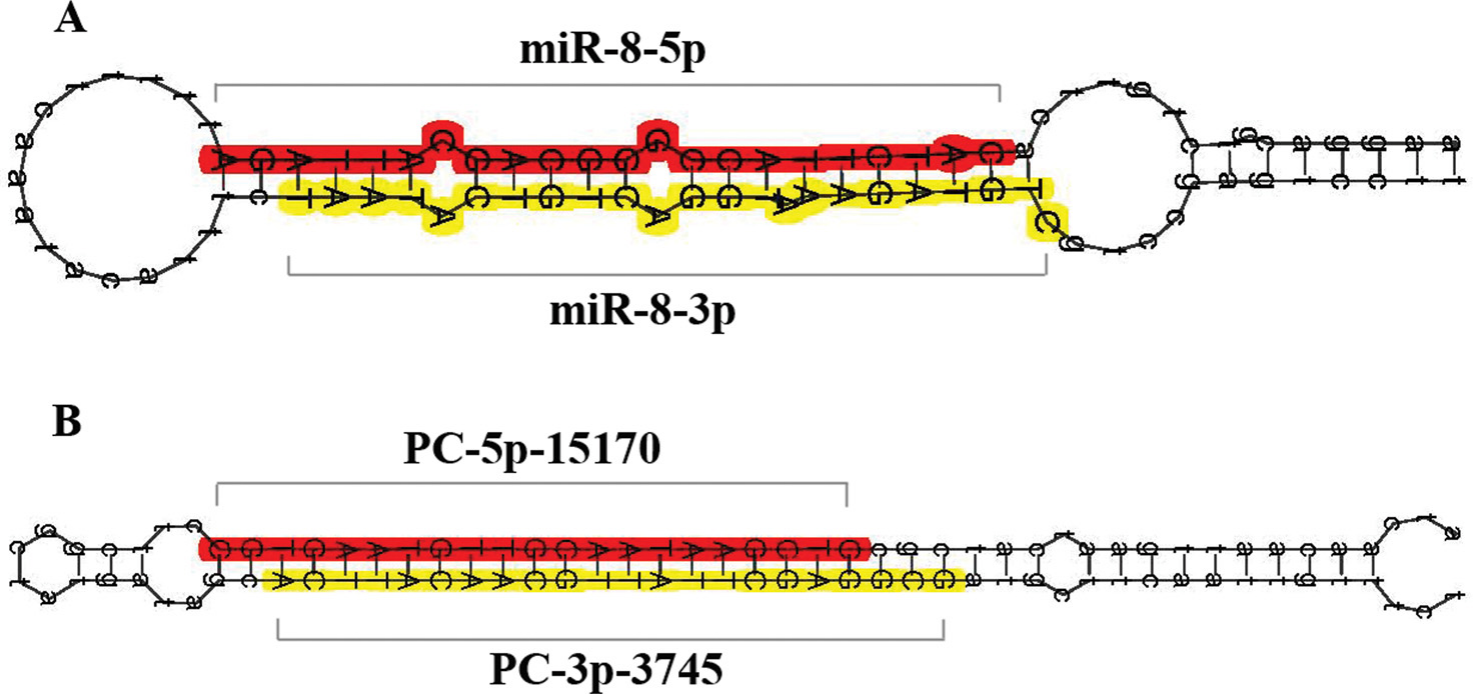
MiRNA hairpins. (A) The predicted hairpin structure of the miR-8 hairpin, colored as miRNA and miRNA*. Red reflects miR-8-5p and yellow reflects miR-8-3p. (B) The predicted hairpin structure of the novel candidate hairpin, colored as miRNA and miRNA*. Red reflects PC-5p-15170 and yellow reflects PC-3p-3745.

In addition to the known miRNAs, the remaining sequences were aligned with the whole genome sequence (WGS) of *B*. *dorsalis* to identify novel miRNAs. Sequences that did not mapped to the conserved sequences in miRBase 20.0 and previously identified known miRNAs of *B*. *dorsalis* were considered as novel or species-specific miRNAs. Using MIREAP software (http://sourceforge.net/projects/mireap/), 120 new novel miRNAs were obtained in the four libraries altogether (S2 Table). Amongst these novel miRNAs, 74, 74, 112 and 94 were detected in the females, males, ovaries and testes, respectively. Notably, almost all these novel miRNAs are either -5p or -3p arms of proposed precursors, which is coordinated with previous reports [25, 26]. The precursor secondary structure of each novel miRNA was constructed and the negative-folding free energies of their secondary structures range from 63.4 to 15.0 kcal/mol (S2 Table). In addition, secondary structures of one known and one novel miRNAs are shown in Fig 2.

### Characterization of known and novel miRNAs

**Fig 3:**
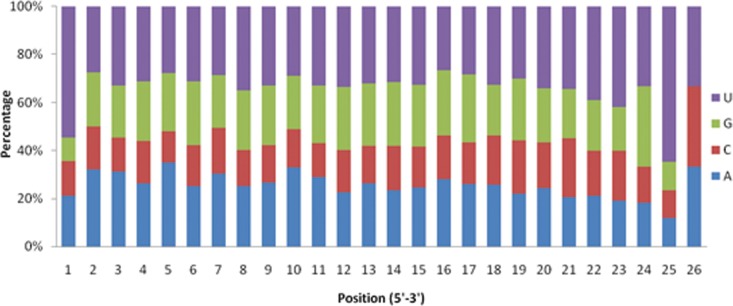
First nucleotide bias in different sites from 1 to 26 (5′-3′) in *B*. *dorsalis* miRNAs.

Base composition can affect the physiochemical and biological properties of miRNA including the secondary structures of miRNAs and the activity of enzymes [27,28]. Analysis of the nucleotide bias in miRNAs have been proved a dominant base for uracil (U) at the first nucleotide [29,30]. In our study, we analyzed the first nucleotide bias in all identified miRNAs. The percentage of the four nucleotides at first nucleotide showed that U was frequently existed (54.48%) at the 5’ end (Fig 3). The nucleotide bias of miRNAs in insects indicated this phenomenon might be involved in the regulation of the target gene.

**Fig 4:**
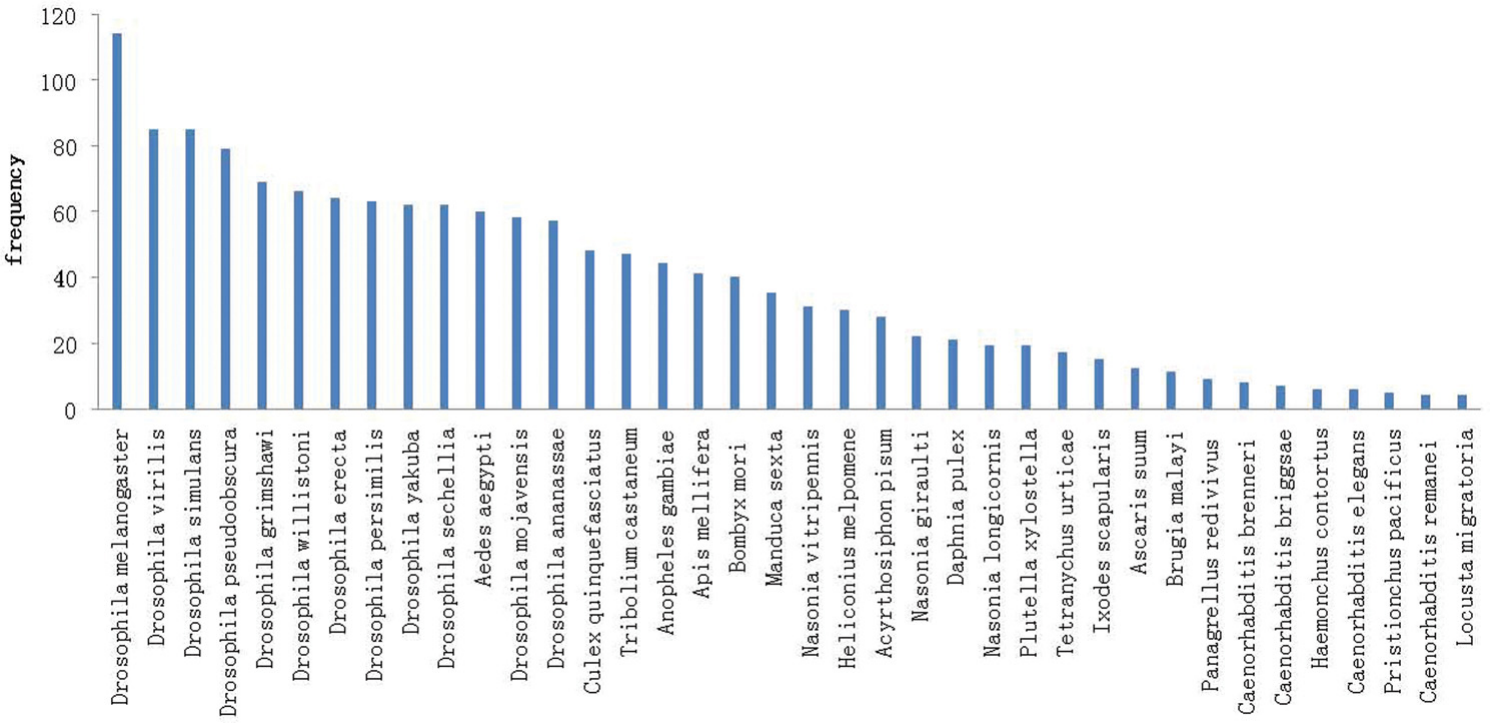
Conserved miRNAs in *B*. *dorsalis* against miRNAs of other species.

We identified a total of 303 miRNAs, of which 183 known miRNAs belonged to 91 families. The conservation profile of the identified miRNAs that matched to other species are presented in Fig 4. Those conserved miRNA families partake the same seed regions and a few different nucleotide in the non-seed sequence regions. The seed sequence (bases 2–8 from the 5’ end) plays a key role in miRNA-target recognition for translational inhibition or mRNA cleavage [31]. The seed region ATCACAG in our study comprised 23 unique miRNAs including miR-2a, miR-2b, miR-5, miR-6, miR-11, miR-13a, miR-13b, miR-308 and miR-994 which belong to the same families demonstrate that miRNAs shared the same seed can regulate the gene expression corporately in different biological processes.

### Sex-biased expression of miRNAs

**Fig 5:**
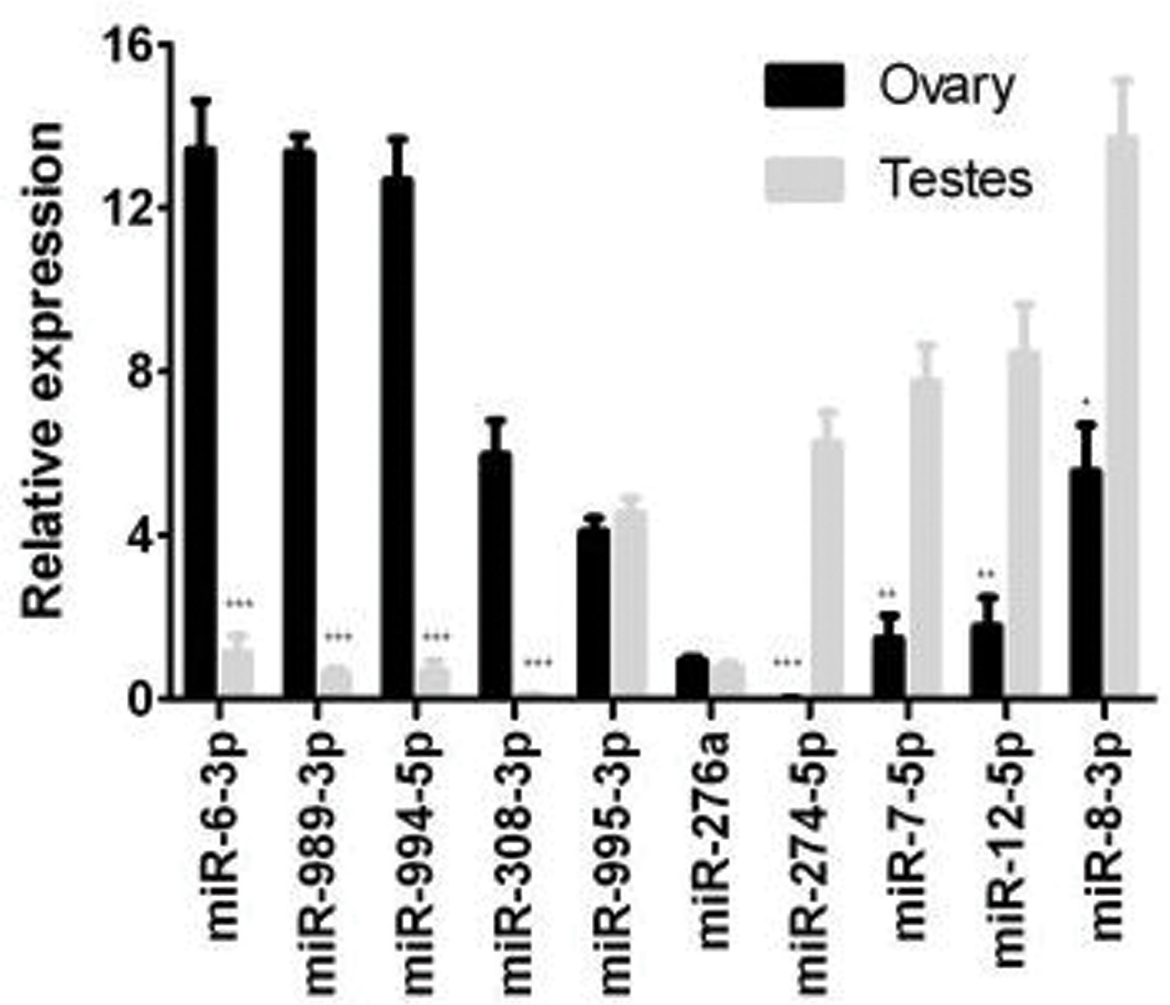
Real-time quantitative PCR validates expressions of 10 known miRNAs. The amount of expression was normalized to the level of α-tubulin. Error bars show SD from three independent experiments with three triplicates each and asterisks (* or** or ***) indicate significant differences (P< 0.05 or P<0.01 or P<0.001, respectively) compared to the relevant control in a two-tailed t-test.

To characterize the sex-biased expression patterns in *B*. *dorsalis*, the miRNAs from females, males, ovaries and testes library were cross-compared. In our study, we identified 18 female-biased and 16 male-biased miRNAs. Of the sex-biased miRNAs, some are pairs derived from the same precursor (like miR-5-5p and miR-5-3p; miR-8-5p and miR-8-3p), and most of the miRNAs prefer to cluster in the genome and express in a consistent sex-biased pattern (Table 2). Intriguingly, the female-biased and male-biased miRNAs are highly expressed in ovary and testes respectively (S3 Table). This suggests that sex-biased miRNAs are mainly involved in the reproductive function. qRT-PCR experiments were used to validate deep sequencing data by measuring the expression profiles of 10 random selected miRNAs. The results showed that miR-6-3p, miR-989-3p, miR-994-5p, miR-308-3p were significantly more highly expressed in the ovaries, miR-8-3p, miR-1-3p, miR-12-5p, miR-274-5p was preferentially expressed in the testes, while miR-995-3p and miR-276a co-expressed in the ovary and testes (Fig 5). The consistency of the miRNA expression and deep sequencing data indicates that all the analysis in our study is reliable.

**Table 2:** MicroRNAs with sex-biased expression and genome location.

female-biased				male-biased:			
miR-996-3p	JFBF01000218.1	miR-306-5p	JFBF01000027.1	miR-8-5p	JFBF01000093.1	miR-999-3p	JFBF01000069.1
miR-994-5p	JFBF01000147.19	miR-263a-5p	JFBF01000022.1	miR-8-3p	JFBF01000093.1	miR-276b-3p	JFBF01000015.1
miR-318-3p	JFBF01000147.1	miR-125-5p	JFBF01000010.1	miR-3477-5p	JFBF01000073.1	miR-34-5p	JFBF01000004.1
miR-308-3p	JFBF01000075.1	miR-9a-5p	3L	miR-12-5p	JFBF01000073.1	miR-1-3p	JFBF01000002.1
miR-10-5p	JFBF01000063.1	miR-92b-3p	3R	miR-275-3p	JFBF01003687.1	miR-1000-5p	3R
miR-309-3p	JFBF01000054.1	miR-5-5p	2R	miR-7-5p	JFBF01000368.1	miR-252-5p	3R
miR-6-3p	JFBF01000054.1	miR-5-3p	2R	miR-987-5p	JFBF01000144.1		
miR-4-3p	JFBF01000054.1	miR-989-3p	2R	miR-274-5p	JFBF01000088.1		

### Target prediction for sex-biased miRNAs and function analysis

**Fig 6:**
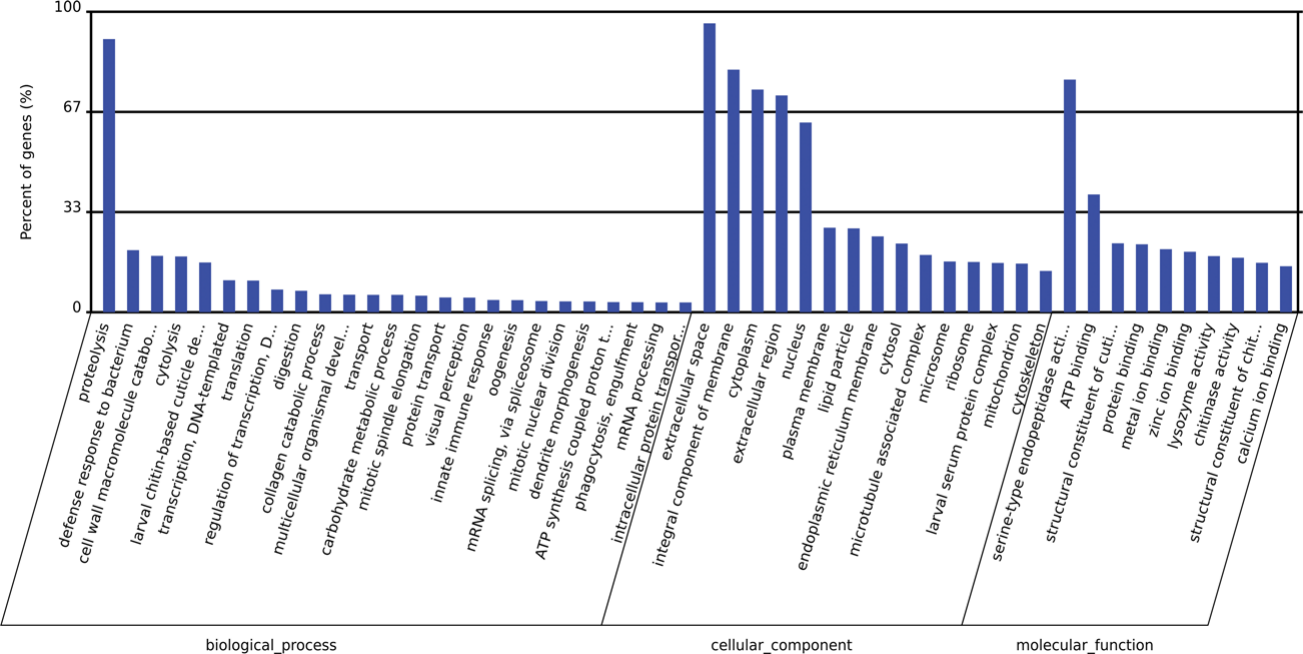
GO classifications of unigenes derived from sequencing miRNAs in *B*. *dorsalis*. The terms were summarized into 3 main GO categories and 50 sub-categories. The y-axis represents the percentage of unigenes.

Intending to understand the physiological functions and biological processes of sex-biased miRNAs in sex determination and gonad differentiation, we annotated the miRNAs and miRNA targets by Gene Ontology (GO) enrichment and Kyoto Encyclopedia of Genes and Genomes (KEGG) pathway analysis. Target prediction was performed by integrating miRanda [32] and TargetScan [33] data. Four female-biased and four male biased miRNAs and their related target gene are listed in Table 3. One target gene of the female-biased miR-989-3p is *doublesex (dsx)* which is considered as the key switch gene in the sex determination of tephritid insects. GO annotation and KEGG pathway analysis were performed to identify functional modules regulated by these miRNAs. GO enrichment analyses of the percentage of genes involved in biological process, cellular component, and molecular component are shown in Fig 6. The KEGG pathway analysis revealed 250 pathways that were enriched with miRNA targets (data not shown). Neuroactive ligand-receptor interaction, PPAR signaling pathway, gamma-Hexachlorocyclohexane degradation and D-Glutamine and D-glutamate metabolism ranked among the most enriched pathways. The 10 most-enriched Go categories for the target genes of miRNAs and KEGG pathways enriched in target genes of miRNAs are shown in S4 Table and S5 Table.

**Table 3:** Potential target gene of sex-biased miRNAs in *B*. *dorsalis*.

miRNA		miRNA sequences (5’-3’)	Target gene	Gene annotation	TargetScan	miRanda	Total
miR-989-3p	UGUGAUGUGACGUAGUGG		TRPL	Transient-receptor-potential-like protein	1	1	2
			CU01	Cuticle protein	1	1	2
			ACOD	Acyl-CoA desaturase	1	1	2
			DSX	doublesex	1	1	2
miR-994-5p	UAAGGAAAUAGUAGCCGUGAUU		PP1B	Serine/threonine protein phosphatase	1	1	2
			TRYE	Trypsin epsilon	1	1	2
			CP6A2	Cytochrome P450	1	1	2
miR-5-3p	UAUCACAGUGAUUUUCCUUGU		SP24D	Serine protease	1	1	2
			RERGL	Ras-related and estrogen-regulated growth inhibitor-like protein	1	1	2
miR-318-3p	UCACUGGGCUUUGUUUAUCUCA		ATPB	ATP synthase	1	1	2
			TITIN	Titin	1	1	2
			RRP44	Exosome complex exonuclease	1	1	2
miR-8-5p	CAUCUUACCGGGCAGCAUUAGA		RENI1	Renin-1	1	1	2
			PGK	Phosphoglycerate kinase	1	1	2
			URM1	Ubiquitin-related modifier 1 homolog	1	1	2
			HSBP1	Heat shock factor-binding protein	1	1	2
miR-252-5p	CUAAGUACUAGUGCCGCAGGACU		YTV2	Uncharacterized zinc metalloprotease	1	1	2
			SNMP1	Sensory neuron membrane protein	1	1	2
			LPH	Lactase-phlorizin hydrolase	1	1	2
miR-274-5p	UUUGUGACCGACACUAACGGGU		TRY	Trypsin	1	1	2
			MDR49	Multidrug resistance protein	1	1	2
			NLTP	Non-specific lipid-transfer protein	1	1	2
miR-12-5p	UGAGUAUUACAUCAGGUACUGGU		SER1	Serine proteases	1	1	2
			MTH11	G-protein coupled receptor	1	1	2
			COQ6	Ubiquinone biosynthesis monooxygenase	1	1	2

## Discussion

To explore the differential expressed miRNAs between sexes and gonads, we identified and characterized miRNAs from females, males, ovaries and testes in *B*. *dorsalis* by Solexa deep sequencing. A total of 183 known and 120 novel miRNAs were gained from the four libraries. The length distribution of the total small RNA reads in data set presented that the dominant size was 21 nt followed by 22 and 20 nt sequences which is the typical length of the mature miRNAs, but a very low in 24, 25, 26 nt. In the previous report, only a total of 123 known and 60 novel miRNAs were identified in *B*. *dorsalis* [25]. The available of the whole genome sequence (WGS) of *B*. *dorsalis* enlarge the finding of known and novel miRNAs in our study.

Among these miRNAs in our study, 44 pairs of known miRNAs and miRNA*s (postfixed with 3p and 5p) were observed. The miRNA and miRNA* sequences from 44 pairs either share similar or differential expression. miRNA and miRNA* are the products of the same pre-miRNA and functioned differently [34]. miRNA is stable and loaded into RISC to recognize the target gene, while miRNA* is considered inactive and degraded immediately in cytosol [35,36]. But the recent reports showed that some miRNA* with high expression play potential roles in regulation [37]. The 3’ and 5’ arm miR-10 are co-expressed and both target *Hox* genes in Drosophila [38–40]. These miRNA precursors may produce functional molecules from both arms. In fact, miRNA*s could have some endogenous targets; Okamura found that *D*. *melanogaster* miR-iab-4-3p can involved in endogenous regulatory networks by target *abrupt* gene [41]. This differential expression of the 3p or 5p miRNA from the both arms of precursors are associated with specific tissue [42], and indicate a clue in miRNA evolution [43].

In our study, we identified 91 families from the 183 known miRNAs. miR-2a, miR-2b, miR-5, miR-6, miR-11, miR-13a, miR-13b, miR-308 and miR-994 with same seed region ATCACAG in our study is the most abundant family. miR-2 (miR-2, miR-13a and miR-13b) is involved in the metamorphosis through repressing the *Krüppel* homolog 1 (*Kr-h1*) expression in *Blattella germanica* [44]. While in Drosophila, miR-2 control apoptosis by targeting potential proapoptotic genes (*reaper*, *grim* and *sickle*) [45]. miR-10 members are considered a close interplay in regulation *Hox* [46]. All these signs show that miRNA family is conserved among species, but can alter target gene via different seed sequences during the course of evolution [47]. Previous studies showed that the first and ninth nucleotide which limit the seed region at the 5’ end of mature miRNA are enriched with uridine (U) and vital to miRNA-target recognition [31]. In our study, we found that uridine (U) was the most abundant (54.48%) at first nucleotide but not at ninth nucleotide, which is coordinated with previous reports [25].

In *D*. *melanogaster*, amounts of female-biased and male-biased miRNAs have found and distributed mostly in the gonad [4]. It is reported that these sex-biased miRNAs are encoded within the sex-biased genes and associated with the reproductive function [43,48]. Our study obtained 18 female-biased miRNAs and 16 male-biased miRNAs. Among these miRNAs, miR-989-3p, miR-994-5p, miR-996-3p, miR-318-3p, miR-92b-3p, miR-306-5p, miR-12-5p were also sex-biased expression in *D*. *melanogaster* [4], indicating the conservation of those sex-biased miRNAs among different species. miR-5-3p/miR-5-5p, miR-4-3p/miR-6-3p/miR-309-3p, miR-318-3p/miR-994-5p from female-biased miRNAs and miR-8-5p/miR-8-3p, miR-3477-5p/miR-12-5p, miR-252-5p/miR-1000-5p from male-biased miRNAs are clustered in *B*. *dorsalis* genome, suggesting that those clustered miRNA in *B*. *dorsalis* is transcribed in multicistronic primary-miRNA. Unexpectedly, all the sex-biased are located in the autosome rather than the male-biased miRNAs emerged in the X chromosome as reported [4]. The X chromosome cluster miR-982-5p, miR-983-5p, miR-984-5p, miR-973-3p, miR-9369-5p, miR-4966-5p, miR-975-5p in our library have a low expression in all samples, which is different from previous reports in *D*. *melanogaster*, the enriched X chromosome cluster miR-982/983/984 and miR-973/4966/975 are highly expressed in testes of *D*. *melanogaster* [4]. The divergence of X chromosome cluster miRNAs between *D*. *melanogaster* and *B*. *dorsalis* may be the results of a lossment of male genes and male-biased miRNAs from the X chromosome in evolution [4,49].

By using miRanda and TargetScan software, the target genes of sex-biased and gonad-specific miRNAs were predicted. We found one target gene of the female-biased miR-989-3p is *doublesex (dsx)* which is considered as the key switch gene in the sex determination of *Bactrocera* pests. The primary signal of sex-determination cascade in the tephritid insects relies on a Y-linked male-determining factor (M-factor) [50], while *dsx* is the bottom gene of the cascade and conserved in the regulation of sexual differentiation [51,52]. The linkage between miR-989-3p and *dsx* in our study indicate that sex-biased miRNAs may not only associated with the reproductive function but also involved in the sex-determination by regulating the cascade genes in *B*. *dorsalis*. Fagegaltier et al. (2014) found that *let-7* as a ecdysone-mediated signaling factor affect the expression of sex-specific genes including *sexlethal* (*sxl*), *dsx* and yolk protein gene (*yp1*), which to some extent, maintaining sexual identity in the soma of *D*. *melanogaster*. Also, there are no miRNAs on the Y chromosome in *B*. *dorsalis*. Whlie in *Bombyx mori*, a W-chromosome specific small RNA (piRNA) have an effect on the sex-specific *dsx* splice variants and determined the primary sex in the WZ sex determination system [53]. The deficiency of Y-chromosome miRNAs in Diptera species indicates a different sRNA-mRNA interplay mechanism in XY system.

In this study, the potential miRNAs in females, males, ovaries and testes of *B*. *dorsalis* were identified and characterized using deep sequencing, and 183 known miRNAs, 120 novel miRNAs were abtained. The different expression between sexs and gonads suggested that these miRNAs may play important roles in reproduction processes such as spermatogenesis and oogenesis. Further function analyses of the sex-biased miRNAs and their target genes will be of great significance to better understand the somatic and germline sex determination mechanism in *B*. *dorsalis* and to develop new control approaches by miRNA interruption.

## Materials and Methods

### Sample preparation and RNA extraction

*B*. *dorsalis* were reared on artificial diet and cultured at 28°C under a photoperiod of 12 h light:12 h dark described previously in our laboratory [54]. Newly emerged virgin females and males were separated by sex and after 12 days mature female and male adults were dissected. After dissection, the gonads and the rest adult samples were immediately stored in RNAlater^®^ Solution (Ambion). To identify the small RNAs involved in *B*. *dorsalis* gonad proliferation and sex differentiation, four small RNA libraries were constructed from the mature adults (without ovaries and testes respectively) and gonads samples. Total RNAs were extracted using Trizol reagent (Invitrogen, CA, USA) according to the manufacturer’s protocol, the quantity and purity were examined using an Bioanalyzer 2100 (Agilent, CA, USA).

### Small RNA library construction and deep sequencing

Small RNA libraries were generated from the four RNA samples with the TruSeq Small RNA Sample Prep Kits, following manufacturer’s guide (Illumina, San Diego, USA). The 18–30 nt length RNA fractions were separated by gel and ligated sequentially to 3’ and 5’ adaptors. Ligation products were then reverse transcribed using the primer on the 3’ adaptor and 15 PCRs amplified on the first strand synthesis production with both adaptor sequence primers. Ultimately, PCR products were purified, validated and sequenced by LC-Sciences using an Illumina Hiseq2500.

### Small RNA sequence bioinformatic analysis

After removing low quality reads and adaptor sequences from the raw datas, large amounts of clean small RNAs were analyzed by BLAST against *B*. *dorsalis* mRNAs database [24], RFAM and Repbase to identify possible mRNA, rRNA, tRNA, snRNA, snoRNA and other ncRNAs. The remaining clean reads were aligned to the miRNA precursors/mature miRNA sequences in miRBase and previously identified miRNAs of *B*. *dorsalis* [25], with the matched or one mismatch sequences, were identified as known miRNAs. The unmapped sequences after known miRNA identification were aligned to the genome of *B*. *dorsalis* and the hairpin RNA structures containing sequences were predicated from the flank 80 nt sequences using RNAfold software complying with criteria from the pre-miRNA statistics in miRBase to identify potentially novel miRNAs [55].

### Comparison of differential miRNAs

The expression patterns of miRNAs between female and male, female and ovary, male and testes were compared in order to identify sex-biased and gonad specific expressed miRNAs. The abundance of each miRNA in the libraries was transformed through a modified global normalization according to the procedures described before [25]. Each library miRNAs expression were normalized to transcripts per million and those miRNAs which normalized expression were 0 were modified to 0.01. After removing the normalized expression of miRNAs which were less than 1 in four libraries, the normalized data were used to calculate fold-change values [= log_2_(X/Y)] and the log_2_-ratio plot. The statistically significant difference (threshold of a fold change >2 and P-value < 0.05) of each library was tested with Fisher test and chi-square test.

### miRNA target gene prediction and pathway analysis

According to the procedures described in previous work [55], which provided by LC Sciences Service, we predicted the target genes of highly expressed miRNAs and analyzed the GO terms and KEGG Pathway of these miRNAs. First, TargetScan and MiRanda softwares were applied to identify miRNA binding sites. Then we combined the data which predicted by both softwares and calculated the overlaps. The gene ontology (GO) terms and the Kyoto Encyclopedia of Genes and Genomes (KEGG) pathway of these highly miRNAs and miRNA targets were annotated [56] and analyzed with the DAVID Bioinformatics Resources Database [57]. The molecular function, cell component, and biological process were used to organize the target genes by functional classification.

### qPCR assay of known miRNAs

The expression profiles of ten known miRNAs were investigated in this study. Reverse transcription reactions for mature miRNAs and α-tubulin as a control were conducted with three biological replicates total RNA (same RNA sources that were used for the sequencing experiments) using miRNA-specific stem-loop primers or an α-tubulin gene reverse primer. The stem-loop RT primers and gene-specific primers were designed according to previous work [25]. All primers are listed in S6 Table. The reaction system (20 μl) contained 1 μg RNA, 2 pmol stem-loop RT primer or α-tubulin gene reverse primer, 4 μl 5 ×PrimeScript Buffer, 10 nmol deoxynucleotide triphosphates (dNTPs), 20 U Rnase Inhibitor, 100 U PrimeScript Reverse Transcriptase (TaKaRa, Dalian, China) and RNase-free water. The 10 μL reactions consisting of total RNA, stem-loop RT primer or α-tubulin gene reverse primer, 0.25 mM each of dNTPs and RNase-free water were first incubated for 5 min at 65°C, placed in an ice bath for 2 min, briefly centrifuged, and then other reagents were added. The 20 μL reactions were incubated in a MyCycler Thermal Cycler (Bio-Rad, Hercules, CA) for 60 min at 42°C for 15 min at 70°C, and then at 4°C for subsequent processes. The reverse transcription products were used for real-time qPCR. qPCRs were performed using SYBR Green qPCR mix following the manufacturer’s instructions in a real-time thermal cycler (Bio-Rad, Hercules, CA, USA). The qRT-PCR program was as follows: 95°C for 10 min, 40 cycles of 95°C for 15 s, 60°C for 30 s and 72°C for 30 s. Three biological and technical replicates were performed. Real-time expression data were analyzed by 2^-△△Ct^ method [58].

## Supporting Information

S1 Fig Length distribution and abundance of small RNAs in the four libraries. (TIF)

S1 Table Identification of known microRNAs in the four libraries. (XLSX)

S2 Table Identification of combined novel microRNAs in the four libraries. (XLSX)

S3 Table Identification of sex-biased microRNAs in the four libraries. (XLSX)

S4 Table The 10 most-enriched Go categories for the target genes of miRNAs. (DOCX)

S5 Table The most enriched KEGG pathways for target genes of miRNAs. (DOCX)

S6 Table Primers used in our study. (XLSX)
