# Supplementary figures and images for: Identification and Characterization of Sex-Biased MicroRNAs in Bactrocera dorsalis (Hendel)
Source: PLoS One. 2016 Jul 21;11(7):e0159591. doi: 10.1371/journal.pone.0159591 (PMC4956098; doi:10.1371/journal.pone.0159591)

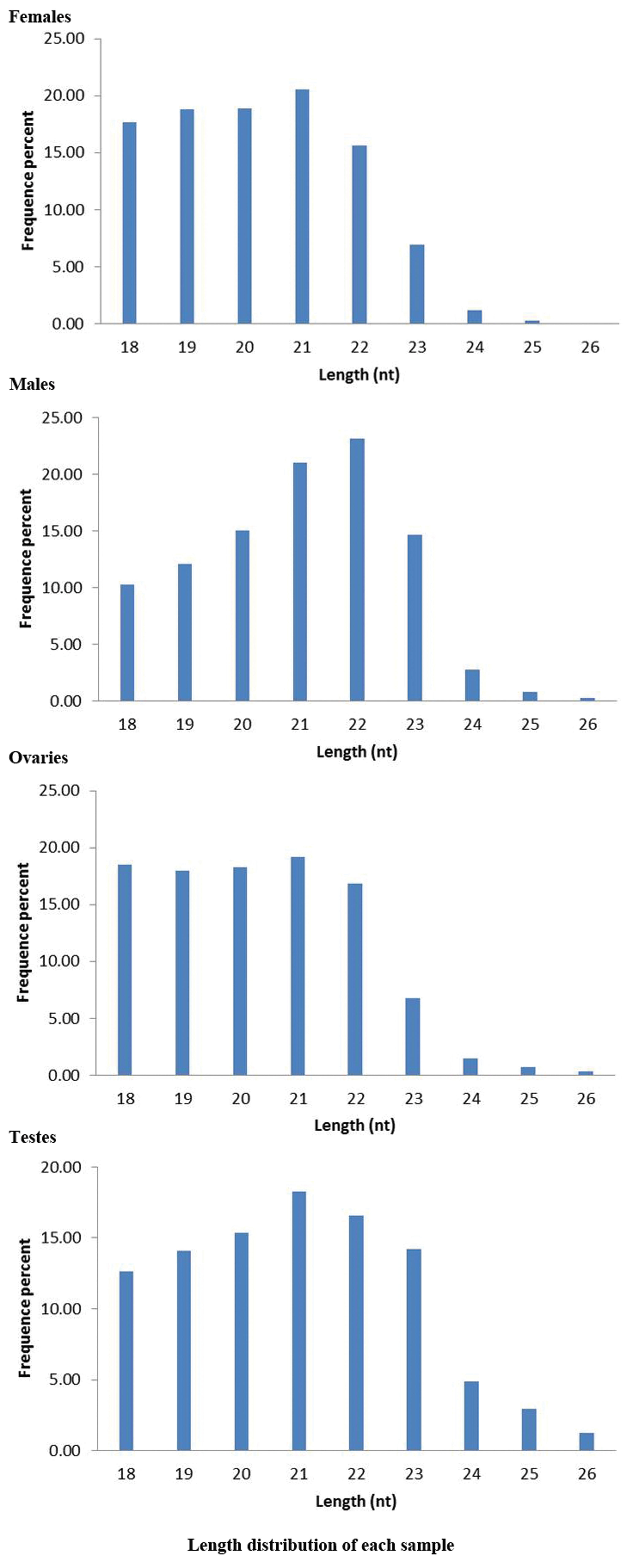

Supplement: S1 Fig — (TIF) [file pone.0159591.s001.tif]
