# Supplementary material for: Identification and Characterization of Sex-Biased MicroRNAs in Bactrocera dorsalis (Hendel)
Source: PLoS One. 2016 Jul 21;11(7):e0159591. doi: 10.1371/journal.pone.0159591 (PMC4956098; doi:10.1371/journal.pone.0159591)
Supplement: S4 Table — (DOCX) [file pone.0159591.s005.docx]

| GO ID | GO terms | S-gene  number | TS-gene  number | B-gene  number | TB-gene number | P-value |
| --- | --- | --- | --- | --- | --- | --- |
| A: Biological process | | | | | | |
| GO:0008363 | larval chitin-based cuticle development | 610 | 15,541 | 925 | 42,736 | 5.67E-76 |
| GO:0006869 | lipid transport | 109 | 15,541 | 156 | 42,736 | 1.78E-17 |
| GO:0051281 | positive regulation of release of sequestered calcium ion into cytosol | 23 | 15,541 | 27 | 42,736 | 2.47E-07 |
| GO:0014009 | glial cell proliferation | 23 | 15,541 | 27 | 42,736 | 2.47E-07 |
| GO:0046889 | positive regulation of lipid biosynthetic process | 23 | 15,541 | 27 | 42,736 | 2.47E-07 |
| GO:0031999 | negative regulation of fatty acid beta-oxidation | 22 | 15,541 | 26 | 42,736 | 5.82E-07 |
| GO:0031670 | cellular response to nutrient | 22 | 15,541 | 26 | 42,736 | 5.82E-07 |
| GO:0030157 | pancreatic juice secretion | 24 | 15,541 | 30 | 42,736 | 1.29E-06 |
| GO:0008610 | lipid biosynthetic process | 25 | 15,541 | 33 | 42,736 | 4.64E-06 |
| GO:0032228 | regulation of synaptic transmission, GABAergic | 22 | 15,541 | 28 | 42,736 | 6.28E-06 |
| B: Cellular component | | | | | | |
| GO:0005576 | extracellular region | 1,329 | 15,541 | 3,040 | 42,736 | 0.00E+00 |
| GO:0000275 | mitochondrial proton-transporting ATP synthase complex, catalytic core F(1) | 66 | 15,541 | 75 | 42,736 | 2.18E-20 |
| GO:0005616 | larval serum protein complex | 302 | 15,541 | 620 | 42,736 | 1.65E-10 |
| GO:0005615 | extracellular space | 1,771 | 15,541 | 4,534 | 42,736 | 3.78E-05 |
| GO:0008021 | synaptic vesicle | 53 | 15,541 | 93 | 42,736 | 4.05E-05 |
| GO:0043292 | contractile fiber | 24 | 15,541 | 35 | 42,736 | 1.08E-04 |
| GO:0001651 | dense fibrillar component | 7 | 15,541 | 7 | 42,736 | 8.40E-04 |
| GO:0005811 | lipid particle | 514 | 15,541 | 1,273 | 42,736 | 1.47E-03 |
| GO:0030426 | growth cone | 16 | 15,541 | 24 | 42,736 | 2.47E-03 |
| GO:0034362 | low-density lipoprotein particle | 8 | 15,541 | 10 | 42,736 | 6.32E-03 |
| C: Molecular function | | | | | | |
| GO:0042302 | structural constituent of cuticle | 845 | 15,541 | 1,794 | 42,736 | 0.00E+00 |
| GO:0008010 | structural constituent of chitin-based larval cuticle | 606 | 15,541 | 913 | 42,736 | 3.95E-77 |
| GO:0030547 | receptor inhibitor activity | 73 | 15,541 | 85 | 42,736 | 4.98E-21 |
| GO:0000062 | fatty-acyl-CoA binding | 144 | 15,541 | 221 | 42,736 | 3.06E-18 |
| GO:0045735 | nutrient reservoir activity | 365 | 15,541 | 789 | 42,736 | 5.62E-09 |
| GO:0004736 | pyruvate carboxylase activity | 27 | 15,541 | 33 | 42,736 | 1.14E-07 |
| GO:0004032 | alditol:NADP+ 1-oxidoreductase activity | 31 | 15,541 | 40 | 42,736 | 1.32E-07 |
| GO:0030156 | benzodiazepine receptor binding | 22 | 15,541 | 26 | 42,736 | 5.82E-07 |
| GO:0005344 | oxygen transporter activity | 394 | 15,541 | 892 | 42,736 | 7.76E-07 |
| GO:0046961 | proton-transporting ATPase activity, rotational mechanism | 126 | 15,541 | 247 | 42,736 | 1.72E-06 |

Table S4. The 10 most-enriched Gocategories for the target genes of miRNAs (P < 0.05)
