# Supplementary material for: Identification and Characterization of Sex-Biased MicroRNAs in Bactrocera dorsalis (Hendel)
Source: PLoS One. 2016 Jul 21;11(7):e0159591. doi: 10.1371/journal.pone.0159591 (PMC4956098; doi:10.1371/journal.pone.0159591)
Supplement: S5 Table — (DOCX) [file pone.0159591.s006.docx]

| Pathway ID | Pathway description | S-gene  number | TS-gene  number | B-gene  number | TB-gene  number | P-value |
| --- | --- | --- | --- | --- | --- | --- |
| ko04080 | Neuroactive ligand-receptor interaction | 1,643 | 8,705 | 3,907 | 25,612 | 7.77E-16 |
| ko03320 | PPAR signaling pathway | 255 | 8,705 | 568 | 25,612 | 3.36E-08 |
| ko00361 | gamma-Hexachlorocyclohexane degradation | 372 | 8,705 | 946 | 25,612 | 2.69E-04 |
| ko00471 | D-Glutamine and D-glutamate metabolism | 23 | 8,705 | 37 | 25,612 | 4.21E-04 |
| ko00903 | Limonene and pinene degradation | 363 | 8,705 | 931 | 25,612 | 6.47E-04 |
| ko04330 | Notch signaling pathway | 68 | 8,705 | 144 | 25,612 | 6.74E-04 |
| ko00945 | Stilbenoid, diarylheptanoid and gingerol biosynthesis | 267 | 8,705 | 680 | 25,612 | 2.01E-03 |
| ko04514 | Cell adhesion molecules | 21 | 8,705 | 36 | 25,612 | 2.35E-03 |
| ko05210 | Colorectal cancer | 71 | 8,705 | 162 | 25,612 | 5.72E-03 |
| ko01040 | Biosynthesis of unsaturated fatty acids | 118 | 8,705 | 286 | 25,612 | 5.92E-03 |
| ko04912 | GnRH signaling pathway | 95 | 8,705 | 225 | 25,612 | 5.96E-03 |
| ko04740 | Olfactory transduction | 57 | 8,705 | 127 | 25,612 | 6.89E-03 |

Table S5. The most enriched KEGG pathways fortarget genes of miRNAs (P < 0.05)
